# Analysis of the use of behavioral data from virtual reality for calibration of agent-based evacuation models

**DOI:** 10.1016/j.heliyon.2023.e14275

**Published:** 2023-03-04

**Authors:** Vojtěch Juřík, Ondřej Uhlík, Dajana Snopková, Ondřej Kvarda, Tomáš Apeltauer, Jiří Apeltauer

**Affiliations:** a Department of Psychology, Faculty of Arts, Masaryk University, Brno, Czech Republic; b Institute of Computer Aided Engineering and Computer Science, Faculty of Civil Engineering, Brno University of Technology, Brno, Czech Republic; c Department of Geography, Faculty of Science, Masaryk University, Brno, Czech Republic

**Keywords:** Pathfinder, Virtual reality, Evacuation behavior, Agent modeling, Indoor navigation, Evacuation time

## Abstract

Agent-based evacuation modeling represents an effective tool for making predictions about evacuation aspects of buildings such as evacuation times, congestions, and maximum safe building capacity. Collection of real behavioral data for calibrating agent-based evacuation models is time-consuming, costly, and completely impossible in the case of buildings in the design phase, where predictions about evacuation behavior are especially needed. In recent years evacuation experiments conducted in virtual reality (VR) have been frequently proposed in the literature as an effective tool for collecting data about human behavior. However, empirical studies which would assess validity of VR-based data for such purposes are still rare and considerably lacking in the agent-based evacuation modeling domain. This study explores opportunities that the VR behavioral data may bring for refining outputs of agent evacuation models. To this end, this study employed multiple input settings of agent-based evacuation models (ABEMs), including those based on the data gathered from the VR evacuation experiment that mapped out evacuation behaviors of individuals within the building. Calibration and evaluation of models was based on empirical data gathered from an original evacuation exercise conducted in a real building (N = 35) and its virtual twin (N = 38). This study found that the resulting predictions of single agent models using data collected in the VR environment after proposed corrections have the potential to better predict real-world evacuation behavior while offering desirable variance in the data outputs necessary for practical applications.

## 1 Introduction

Evacuation is typically understood as a systematic movement of a person from a dangerous place to a place of safety, if conducted successfully the evacuee should arrive at designated assembly point. This means that evacuation is considered a human-centered activity and the whole process can be categorized as a path-search or aided wayfinding in case of unfamiliar indoor environments [1]. From this perspective, it is directly determined by individual human cognitive abilities and traits, including decision-making strategies, spatial skills, or aptitudes for understanding indoor environments [[2], [3], [4]]. A wide range of complex behavioral phenomena occur during human evacuation, such as prolonged reaction to fire alarms (pre-evacuation behavior) [[5], [6], [7], [8], [9], [10]] or the frequent use of retracing navigation strategy, whereby inexperienced individuals, in particular, evacuate by the same route they entered the building while failing to notice/follow evacuation signs [5,6,11,12]. People also often use other than the prescribed emergency exits [13]. Evacuation behavior is also influenced by the contextual environmental information that is present at the time [14,15]. Previous studies have analyzed the impact of the presence of navigation aids, signage, and landmarks [5,6,15,16], light [17,18], visual, auditory, or olfactory cues that depend on the form of the threat that triggers the evacuation [[19], [20], [21]], and the affordances proposed by the environmental structure itself [11,22,23]. This has placed behavioral studies of individuals in the specific environment at the center of current research. For effective prediction with regard to building designs, it is necessary to capture and incorporate human factors into evacuation models.

### 1.1 Agent-based models for predicting evacuation

Agent-based evacuation models (ABEMs) are used for various kinds of simulations in order to explore, visualize and predict potential scenarios in evacuating people from buildings. Most commercially used agent models are however limited to direct ways of agent movement. They possess no cognitive or behavioral predictors; they do not usually consider many of the specific cognitive and behavioral manifestations that occur in evacuees [24]. ABEMs usually work on the basis of algorithms that calculate the spatio-temporal optimum of the agent movement (e.g., the shortest distance or shortest time). Although smoothing algorithms offer somewhat more natural courses of movement [6], ABEMs usually neglect the fact that the dynamics and trajectories of human movement in real environments do not fully correspond to simulated ones [25,26]. For ABEMs it is typical that agents have predefined parameters based on which they make decisions and choose their paths. Alternative approach is force-based models (e.g., social force model [27]), where agents adapt their behavior to forces from other agents that affect them and cellular automata, which is a specific form of ABEMs and are discrete in time, space, and state variables. It uses cells and discrete mesh. Every cell has a finite number of states and is updated based on a simple set of rules (interacting with other agents and environment [28]). In this study we worked with the software Pathfinder which uses continuous triangulation which facilitates continuous movement of persons throughout the model, compared to cellular automata.

### 1.2 VR experiments as a data source on evacuation behavior

Virtual reality (VR) experiments represent ecologically valid, controllable [29,30] and potentially cost-effective ways of gathering behavioral data on human actions in a wide range of situations. VR seems to activate brain mechanisms similar to those that occur in the real world [31,32]. In this regard, it has been found to effectively simulate real-world scenarios [33]. Virtual environments provided via immersive head-mounted displays (HMDs) possess a high level of realism and allow for the non-invasive collection of behavioral data, which is further promoted by a controllable degree of virtual environment’s interactivity [34] and activity-logging options [22,35,36]. Regarding this, VR experiments^1^ could solve the problem of conducting complicated and expensive evacuation drills in real buildings to gather the necessary data for model optimizations. Moreover, VR-based experiments can be easily performed in virtual buildings that do not currently exist (e.g., proposed buildings) [18,22,[37], [38], [39]] and can be applied for situations related to dynamic hazards, such as fire or the spread of toxins [40]. In this regard, Building Information Modeling (BIM) technology may be used to promote the effective design of VR environments, following the need for human-centered design assessment [35,41]. Recent advances in VR have shown promise in simulations with regard to viable options for capturing behavioral data about actual human behavior during evacuations [2,20,40,[42], [43], [44], [45]]. However, the reliability and usability of specific behavioral properties in VR-based research remains unclear and needs to be explored in relation to the technical opportunities and limits of VR tools and the specific correspondence of VR-generated experiments to real evacuation behavior. For example, the navigation movement as well as other behaviors in real environments compared to virtual ones are expected to vary based on the specific features of the environments [46,47]. Previous research suggested VR technology to limit human performance [48], taking into account the unnatural "involvement" of users in simulation scenarios [49] imperfect depiction or problematic metaphors of movement [50]. The movement activity, especially, was found to be less effective in the virtual environments [51]. This notion brought us to the creation of hybrid agent models resolving the movement metaphor - see below. Other limitations of VR technology include potential cybersickness, i.e., the discomfort that users may experience while using VR equipment, losses in ecological validity compared to field studies since participants in the experiment may be aware that they are part of a simulation, and technical limitations [40,52,53]. These aspects should be always considered when interpreting the outcomes of VR-based research. In the cognitive processes such as decision or reaction times and choice of exit, however, the assumptions about their similarity in real and virtual environments suggested by previous studies [31,54] seems to be supported [51]. Generally, we can conclude that with an appropriate method, the user studies and especially the data on participant movement and decision-making processes from VR-based experiments may help promote the effectiveness and precision of agent models regarding evacuation predictions, as human factors are fundamental variables in the outcomes of building emergencies [5].

### 1.3 Aims of the study

It was demonstrated that there exist differences between the real evacuation process and the evacuation process conducted in VR condition [51], especially regarding total egress times caused by the different movement metaphor. Regarding this, we suppose that VR evacuation simulations cannot be directly used for making predictions about the evacuation process in a real setting. Human behavior in the VR evacuation exercise, however, possesses several similarities to the real evacuation [51] and we argue that it can be used for calibrating ABEMs with the aim to bring necessary predictions applicable for engineering practice. In this study we explore ways of how the VR evacuation data can be used in ABEMs with the aim to reach predictions about real evacuation behavior. For this purpose, the VR-based evacuation experiment [51] was brought into the context of agent-based evacuation modeling. We adopted raw de-identified data about the human individual evacuation behavior in a real and a corresponding virtual building (digital twin). All the used data were gathered within the experiment conducted under the research project, which was solved by the Masaryk University, Brno, in cooperation with Brno University of Technology, Czech Republic, and its outcomes were reported in a solitary report [51] including also detailed methodology and procedure of the experiment. The conducted experiment primarily focused on comparing the behavioral manifestations of individuals evacuating from a real building and individuals evacuating from the same environment created and presented in VR under equivalent conditions. VR setting in the original experiment employed currently accessible VR and controlling devices (head-mounted display for immersive VR and computer mouse and keyboard) with the aim to approach engineering practice. In the present paper, the raw data from the mentioned VR experiment were re-used as key quantities for the Pathfinder ABEM [55] with the goal to analyze the possibilities of using such data collected in cost-efficient and time-efficient VR experiments for agent models. The evaluation of agent models is problematic, as there is a lack of data on real evacuation for the assessed building. In our case however, we are able to compare the resulting model against the available behavioral data collected in the real building. The aim of this study, though, is not to compare behavior aspects captured in a real and a corresponding virtual context nor the discussion on the most effective VR setting for recording evacuation behavior data. With the use of ABEMs, this study aims to assess the potential viability of VR behavioral data for making reliable evacuation predictions. Model outcomes (more specifically, evacuation times) can be statistically compared to the real evacuation data in order to study reliability of ABEMs calibrated by the VR data and to observe differences and similarities with other calibration settings. Here, an evacuation model was run for a specific building using the default parameters of Pathfinder, and results (total evacuation time) were then compared with the simulation results based on VR and real-world experiment data (real-world data served as a baseline). By default settings we mean initial parameters used in Pathfinder software which are based on empirical data with different context (e.g. Ref. [56]) and cannot consider local behavioral patterns such as retracing strategy or waiting points. This setting (or its variation) would be used in situations where there is no local empirical data about occupants’ behavior and decision-making. Purpose of these settings is to analyze the difference between ABEMs results based on local data (VR experiment data) and general type of empirical data used for evacuation modeling.

## 2 Materials and methods

### 2.1 Software

This study used Pathfinder software (version 2021.2.0525 × 64) [55,57], which was developed by Thunderhead Engineering company. Research has shown that Pathfinder is the most used pedestrian evacuation model in academic and industrial fields [58]. The model consists of three modules: a graphical user interface (GUI), a simulator, and a 3D results viewer. GUI is primarily used to create model geometry and model calibration. Supported input geometry file formats include BIM formats (ifc), 3D formats (dae, fbx) and 2D (dwg, dxf). After the geometry is imported, a triangulated navigation mesh is created. Agents are presented as autonomous entities with a predefined set of parameters [59].

At the beginning of the simulation, for each agent there is an identified local exit in the actual room. Then Pathfinder uses the A* search algorithm [60] for generating seek curves in the navigation mesh [61]. Simulation can be performed in SFPE (Society of Fire Protection Engineers) mode or STEERING mode. SFPE implements the concepts in the SFPE Handbook of Fire Protection Engineering [62]. This mode is based on a flow model, where walking speeds are determined by agent density in each room, and flow through the door is controlled by door width. In this model, agents cannot interact with each other, they can occupy the same space in the geometry and are not able to change their trajectory. In STEERING mode (which was used in our case), agents interact with each other and the environment. Agents use a combination of the steering mechanism (wayfinding) and collision handling to control how they follow their seek curve (their trajectory to the next door, waypoint, etc.) [63,64]. In every time step, the agent's next position is calculated, based on the actual distance from other agents as well as from the surrounding walls, pillars and so on [61]. This makes the agent movement more natural and complex.

### 2.2 Data

**Fig. 1:**
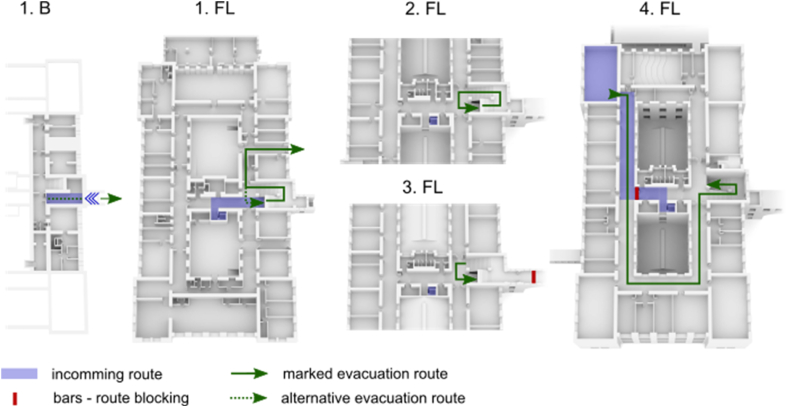
Visualization of the building used in real and VR experiments. Blue is used to mark the incoming route, which was mandatory for each participant, and lead them to the room where the evacuation started. Evacuations routes are marked with green lines, the dashed-line alternative evacuation route (without evacuation signs) leading to the exact exit that was used by participants to enter the building.

Completely anonymized raw data in a form of spatiotemporal coordinates of individual participants movement through the building used for the agent-based models was obtained from the empirical experiment conducted under the research project nr. TL02000103 called “Cognitive psychology and space syntax in virtual environments for agent modeling” investigated by Masaryk University and Brno University of Technology, Czech Republic. The study followed all ethical standards for psychological research, the experiment was conducted according to guidelines of the Declaration of Helsinki. Informed consent was presented to all participants where they were informed about the nature of the experiment, also that their participation was voluntary and that they could leave the experimental session any time they wanted without any negative consequences. In the case of the present study, secondary use of the data was applied. In a between-subjects experimental design, the project aimed to explore the evacuation behavior of users in two different conditions: an evacuation from a real building (35 participants, M = 15, W = 20) and an evacuation from its digital twin - i.e., from an equivalent building presented in immersive virtual reality using head-mounted display (38 participants, M = 22, W = 16). The virtual environment was developed from the source BIM model and further modified in the game graphics engine Unity [41]. The detailed methodology, experiment procedure, and statistical comparisons of the original empirical data on human evacuation behavior in real and virtual environments were presented in a separate project report [51]. In general, in both conditions, the experimental session started in front of the unknown building (see Fig. 1) where participants were one by one instructed to navigate to a specific location in the building - a lecture room. When in the room, participants were distracted with a simple cognitive task (finding differences on two pictures), during whose solution an evacuation alarm sounded so they were forced to evacuate. Since the purpose of the experiment was to explore evacuation behavior and decision-making, participants were prevented from returning by the same route as they entered the building by placing an obstacle in their path (closing bars), which forced participants to look for an alternative evacuation route. During the evacuation, the position in time and gaze activity of the participants were monitored (with the use of cameras and eye-tracking technology in real condition, and automatic logging and embedded eye-tracking in VR condition). An illustration of one participant’s evacuation trajectory is displayed in Fig. 1. From the gathered raw data, we derived detailed behavioral metrics about the participants’ passages through the building (see below). The calculated data were further used to refine the output of the agent evacuation models (Pathfinder [55]).

## 3 Calculations

Taking into account the options of current agent simulation tools, we selected key behavioral metrics obtained from the above-mentioned study [51] to refine Pathfinder ABEM. Navigation movement and other behaviors in real environments, in comparison with virtual ones, are expected to vary based on the specific features of the environments [46,47]. Previous research has suggested that VR technology limits human performance [48] in relation to the unnatural “involvement” of users in simulation scenarios [49], imperfect depiction, or problematic metaphors of movement [49]. The interface metaphor for movement in VR (i.e., the specific settings of movement control in the VR interface such as, for example, a keyboard or joystick) was particularly identified as problematic where a keyboard-mouse control interface was used [51]. Such a setup does not simulate real patterns of changes in speed (accelerations) in avatar movements and only offers either constant speed motion or standing. In cognitive processes such as decision or reaction times and choice of exit, however, the assumptions as to their similarity in real and virtual environments suggested by previous studies [31,54] seems to be supported [30]. In this case, convincing analogies were observed in the analyzed data, either in the overall averages of the measured variables or based directly on similar trends identified via visual analysis.

For refining the VR-based models, we focused on the behavioral parameters that were identified as corresponding in real and virtual environments [51]. The parameters that were selected for the optimization of computed models will be presented in more detail (see Table 1 for a general comparison). They are divided into two groups: general parameters that have the application potential in agent models simulations to run in buildings with the same function but a different spatial configuration, while the second group of parameters is specific for the simulation in our studied building. The Pathfinder model allows us to reflect the distribution of the evacuees (agents) on the basis of gender at the input [59]. Therefore, all the values of input parameters used in this study reflect the specific gender distribution of participants in each experiment and are aggregated on that basis, if not stated otherwise.

**Table 1:** Explicit values of input parameters for the model simulations. (Several values were taken from the data collected in Ref. [22]. For an exact explanation, see the descriptions of the parameters above.)

Input parameters	Default Settings	Real Evacuation (M = 15, W = 20)		Virtual Evacuation (M = 22, W = 16)	
**Gender**	–	43% men	57% women	58% men	42% women
**Pre-evacuation time [s]** location (shape) [min; max]	0 [s]	1,73 (0,38) [3,55; 9,95]	1,65 (0,35) [3; 8,6]	1,82 (0,44) [3.38; 15,72]	1,82 (0,46) [4,23; 14,96]
**Unimpeded walking speed [m/s]** mean (SD) [min; max]	1.19	1.82 (0.278) [1.42; 2.25]	1.78 (0.379) [1.3; 2.68]	2.22 (0.158) [1.96; 2.54]	2.09 (0.223) [1.66; 2.34]
**Staircase walking speed [m/s]** mean [m/s] (coefficient [−])	1.19 (1)	1.294 (0.71)	1.176 (0.66)	2.22 (1)	2.09 (1)
**Acceleration [s]**	1.1	1.1	1.1	0	0
**Wall boundary layer [m]** mean (SD) [min; max]	0.15	0.922 (0.245) [0.559; 1.41]	0.894 (0.279) [0.465; 1.38]	0.91 (0.333) [0.387; 1.53]	0.9 (0.284) [0.372; 1.44]
**Staircase pillars boundary layer [m]** mean (SD) [min; max]	0.15	0.642 (0.183) [0.409; 0.954]	0.647 (0.211) [0.37; 1.00]	0.676 (0.239) [0.29; 1.06]	0.625 (0.242) [0.286; 1.03]
**Decision time in front of the bars** time [s] (distribution [%])	–	0-5 (33) 5-10 (40) 10-15 (7) 15-20 (13) 45-50 (7)	0-5 (30) 5-10 (30) 10-15 (20) 15-20 (5) 20-25 (10) 50-55 (5)	0-5 (50) 5-10 (27.5) 10-15 (5) 15-20 (12.5) 30-35 (5)	0-5 (25) 5-10 (50) 10-15 (13) 15-20 (6) 35-40 (6)
**Decision time in front of the stairs on 4. Floor** time [s] (distribution [%])	–	0 (86.6) 5 (6.7) Try to use elevator (6.7)	0 (95) Try to use elevator (5)	0 (87) Try to use elevator (13)	0 (93.75) Try to use elevator (6.25)
**Decision time in front of the stairs on 1. Floor** time [s] (distribution [%])	–	0-2 (53) 2-4 (26) 4-6 (14) 8-10 (7)	0-1 (50) 2-4 (25) 4-6 (5) 6-8 (20)	0-2 (55) 2-4 (45)	0-2 (68.5) 2-4 (18.5) 4-6 (13)
**Retracing [boolean ]**	–	20.0% yes 80.0% no	30.0% yes 70.0% no	36.4% yes 63.6% no	43.8% yes 56.2% no

### 3.1 General quantities

- *Pre-evacuation time* (Alarm reaction time) [s]: When the alarm goes off in the emergency, people do not start to evacuate immediately, making it the key quantity in the estimation of the evacuation time. However, standard methods in the Czech engineering context as reflected by ČSN (Czech Technical Standards), do not usually consider this parameter [65] The brain must initially process the perceived signal, and depending on the various environmental and individual aspects, there is a delay in the human reaction. Since this delay should be incorporated into artificial agent models, we consider its correction crucial in approaching the behavioral validity of the model. The pre-evacuation time (the time it took the participants to leave the room from the moment the alarm sounded) was set in the model on the basis of observations from Ref. [51]. A log-normal distribution was fitted to this data, which in general corresponds to pre-evacuation time, based on [58].
- *Unimpeded walking speed* [m/s]: Speed was one of the metrics for which it was impossible to ensure comparable conditions between real and virtual environments (because of the above-mentioned movement interface metaphor). In the real environment, the speed of the participants was not limited in any way, while in the virtual environment, it was constant (which is related to the chosen method of movement control: a keyboard-mouse interface). In order to be able to suppress these unwanted differences between environments, we aggregated the time it took participants to go through certain segments of the route (specifically the two longest segments on the 4th floor). Thus, individual shorter stops were also included in the walking time, which in the case of the virtual environment were related to movement control skills. The resulting calculated speed thus came closer to reality. Average speeds were aggregated based on gender for both segments and interpolated with a normal distribution (see Table 1), which served as the model input.
- *Staircase walking speed* [m/s]: The differences between environments with regard to unimpeded walking speed applied to the speed of movement on the stairs, and therefore we followed the same procedure. The participants’ (downward) speed on the staircase was derived from six identical flights of the main staircase. The two farthest measured points of the trajectory were determined on each flight, then the distance between them [m] and the time [s] traveled were calculated. Based on this, an average speed on the given flight of stairs was obtained. Subsequently, the average speeds were aggregated on the basis of gender for all flights of the staircase. After dividing by the average unimpeded walking speed, a coefficient was obtained (see Table 1) by which the unimpeded walking speed of movement assigned to the individual agents was multiplied for the staircase route segments.
- *Acceleration time* [s]: This is the time it takes for the agent to reach maximum speed. This was not possible to determine on the basis of the empirical experiment, so it was estimated according to the default settings. With regard to the features of the VE setting (where the speed is constant and limited by movement controls), the acceleration time was not set for models based on the virtual condition.
- *Wall boundary layer* [m]: During the building walkthrough, agents in models follow the ideal trajectory of movement, i.e., the most spatially effective path. Human movement in buildings does not copy this ideal trajectory. In Ref. [56], Gwynne defines layers between occupant and static objects that can be used for evacuation modeling. Wall boundary layer was measured at identified corners of the building corridors (C1-5 in Fig. 1), for which the minimum avoidance was recorded. A normal distribution with the parameters described in Table 1 was then applied to the aggregated data, reflected by gender.
- *Staircase pillars boundary layer* [m]: The minimum distance participants maintained from the corners of the stairscase pillars was monitored (S1-12 in Fig. 3). The subsequent determination procedure for the model input was identical to that relating to the wall boundary layer.

### 3.2 Building specific quantities

- *Decision time on predefined decision points* [s]: The decision process is a crucial human factor, which has been found to be similar in real and virtual evacuation experiments [51], although it is not usually considered in agent simulations [57]. Since the decision time and the choice of route depend on the specific spatial configuration of the building, we cannot abstract the general parameters applicable for every simulation scenario. Three decision points (points requiring a cognitive assessment of the surrounding situation due to a change in direction, obstacles on the route, etc.) were identified and approximated to polygons on the evacuation route (see Fig. 3). The first decision point (A) was a stop at the bars that blocked the route that the participants had previously used to enter the lecture room. The second stop (B) was the junction on the 4^th^ floor, where it was possible to turn towards the elevator (which it was forbidden to use during the evacuation) or go down the stairs. The third decision point (C) was the point at the bottom of the stairs on the 1^st^ floor, where the paths separated into an officially marked evacuation route and a route returning the same way the participants had previously entered the building. In the study of Stachoň et al. [51], the time [s] that participants spent in these polygons was monitored and used as input for our calculations for the models. In addition, where the participants made a longer deviation from the route at these points (usually towards the elevator), it was manually introduced into the model (see Table 1).
- *Retracing strategy* [boolean]: As with the previous parameter, even retracing depends on the various aspects of the building, although the ratio of people who used the retracing strategy to leave the building was similar in the real and virtual environments [51]. Computed models reflect the retracing behavior of participants by assigning the specific exits to individual agents at the last waiting point on the first floor. The specific distribution is depicted in Table 1.

### 3.3 Evacuation model computation

The Pathfinder agent model at the entrance requires the geometry of the building and allows the setting of input parameters that further distinguish the character of individual agents. Wayfinding is implemented by entering a series of events, at the end of which the building is left by the appropriate exit. Each event takes place based on a specified probability distribution, see Table 1. These events include waiting point selection, walking to waiting points, waiting, and exit selection.

**Fig. 2:**
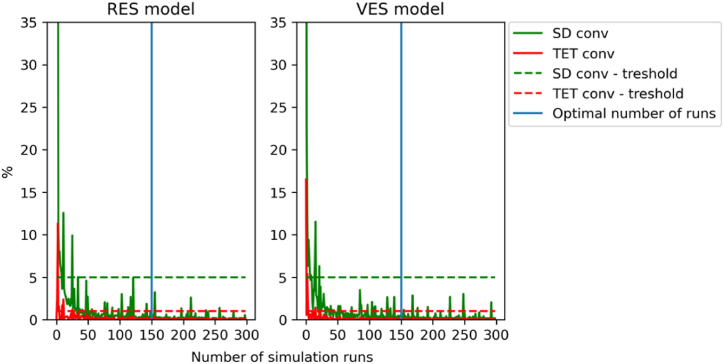
Method of estimation of the minimum required number of simulations based on convergence of total evacuation time and its standard deviation.

The **input data** for the simulations were generated by the Monte Carlo method [66]. In each file, every agent was assigned a unique random number (a so-called random seed) which generated the new parameter values in each simulation within the specified distributions (e.g., movement speed, see Table 1.) for each agent. This mechanism generates variations in the trajectories of the agents and calculated evacuation times, so the outcomes of the simulations could be subjected to statistical analysis. In this study, the number of simulations for the VES model was determined according to Ronchi’s method [67]. Total evacuation time convergence (TET conv) and standard deviation convergence (SD conv) were derived. The other metrics have not been used because they assume the evacuation of a group of people, not an individual. Threshold values were set up for both metrics: TET conv - 1%, SD conv - 5%. 300 simulation test runs were performed for estimation of the minimal required number of simulation runs based on this method (see Fig. 2). Based on the convergence graph in Fig. 2, the number of 150 simulation runs was determined as sufficient for further analysis. It is evident from the graph that a larger number of simulations would no longer affect the variance in the resulting evacuation time. The chosen number of simulations includes all behavioral phenomena defined by the parameters in Table 1, even those that were not so numerous (e.g., trying to use an elevator). In case of models with default settings, the input parameters are used without any distribution, so there is no variability in resulting evacuation times. One simulation is sufficient in this case, however, 35 simulations were performed where the missing variability is seen. Eight examples of these simulations are available in the form of short videos in the Supplementary Materials.

**Fig. 3:**
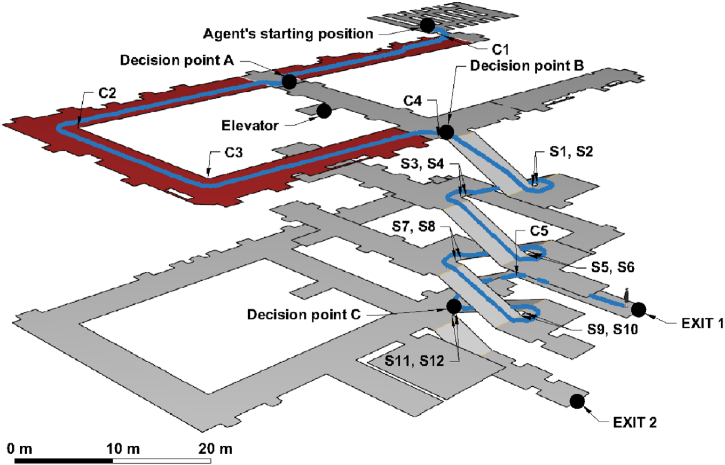
Visualization of the sample evacuation trajectory, important decision points and adjoining corridors. Blue line illustrates the evacuation trajectory. Corridors highlighted in red were used for unimpeded walking speed calculations. Points C1 – C5 represent corridor corners and points S1 – S12 represent staircase pillars.

The key output of the simulations for this study was the total evacuation time, since it is the key and often the only monitored parameter in engineering practice generally considered relevant by investors and local authorities. In this study, evacuation time is the time from the alarm sounding to reaching the exit and is crucial for most evacuation simulations. For this reason, it was selected as an assessment criterion and then became the subject of a comparison of the outputs of the models.

The specific **geometry** of the model was created based on the 3D building documentation (.fbx) created in Revit Architecture (The same model that was used to create the virtual environment). A simplified visualization of the evacuation path and adjoining corridors can be seen in Fig. 3.

### 3.4 Proposed corrections

In the context of this study, we consider evacuation time a fundamental variable for potential application since it represents the key and very often the only monitored parameter in engineering practice. Evacuation time is the principal output of the models, and as it is the key measure in the evacuation predictions. It is primarily dependent on walking speed; however, it is one of the factors where there is no equivalence when we compare the results obtained from empirical VR and real experiment [51] due to different interface metaphors of movement. The input data for this study were taken from a VR experiment using a standard keyboard-mouse control interface, which limits the possibility of acceleration. The participant can either stand or move at a constant speed set at 3 m/s, while the participants egressing the real building were not limited in any way.

Therefore, for the purposes of this study, we applied mathematical corrections to the computed VES model. The first corrected model (Virtual Evacuation Settings Adjusted - VESA model), was calculated using the same input data as for the VES model, except the unimpeded and staircase walking speeds were adopted from the data collected in the real experiment (a real evacuation drill). The second corrected model (Virtual Evacuation Settings Derived - VESD model) was derived from the VES model, in which we corrected the resulting evacuation time by a coefficient calculated as:

$$\frac{total\;average\;walking\;speed\;VE}{total\;average\;walking\;speed\;RE}=\frac{2.184\;m/s}{1.754\;m/s}=1.245$$

**Fig. 4:**
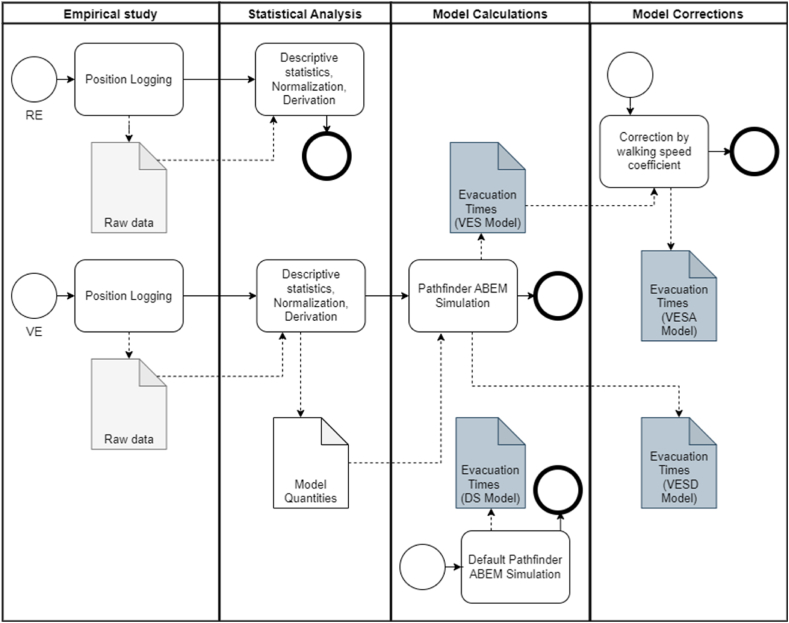
The diagram of model calculation, correction and evaluation and process.

The overall process of model calculations and application of corrections is depicted in Fig. 4.

### 3.5 Models evaluation

The results of the calculated model could be evaluated in four steps. In the first step, we compared the results of the model (VES model) with the model where evacuation simulations took place with default input parameters (DS model). In the second step, we evaluated the model results against empirical data collected in virtual reality (VE). Following the same experiment procedure in both real and virtual environments [51] allowed us to evaluate the model results also against data collected in a real environment (RE). Eventually we conducted the comparisons also for the corrected models. The analysis was conducted using non-parametric ANOVA (Kruskal-Wallis test) and post hoc Dunn’s tests with Holm’s p-value and p-value adjustment for between model comparisons (q-values).

## 4 Results

In this section, the results from the comparison of the computed models are presented. The analysis was conducted using RStudio v. April 1, 1106 [68]. For visualizations, the ggplot2 [69] package was used. The resulting evacuation times from all models were tested for normality using the Shapiro-Wilk test. Since none of them exhibited normal distribution, we chose non-parametric ANOVA (Kruskal-Wallis test) and post hoc Dunn’s tests with Holm’s p-value and p-value adjustment for between model comparisons (q-values).

**Fig. 5:**
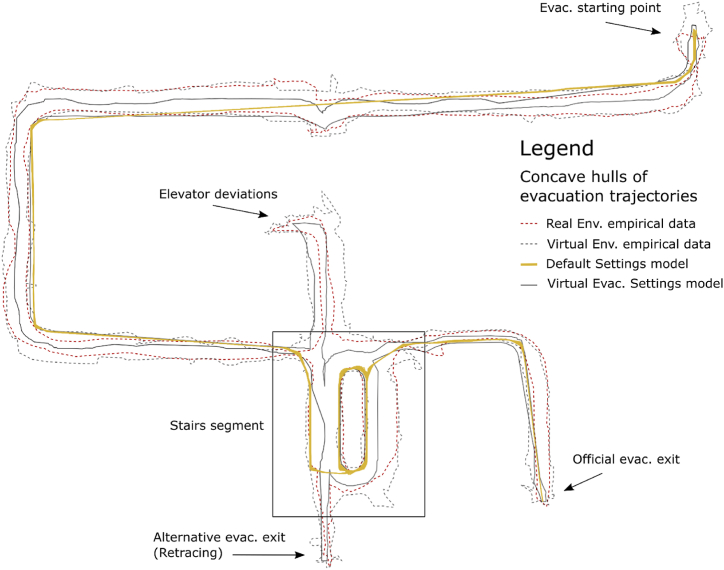
Visual comparison of participant and calculated agent trajectories.

First, we observed that the simulation with default settings generated a different trajectory with no variance. On the contrary, the simulated agent movement in the proposed models overlapped with the envelopes (concave hulls) of real movement trajectories of the participants captured in the empirical experiment (see Fig. 5). The variance in trajectories was introduced by using distributions of model input quantities (see Table 1.) This means that during the simulations, agents of refined models visited similar spaces in the building as real people did, which allows for more detailed interpretations of the reliability of the predictions.

**Fig. 6:**
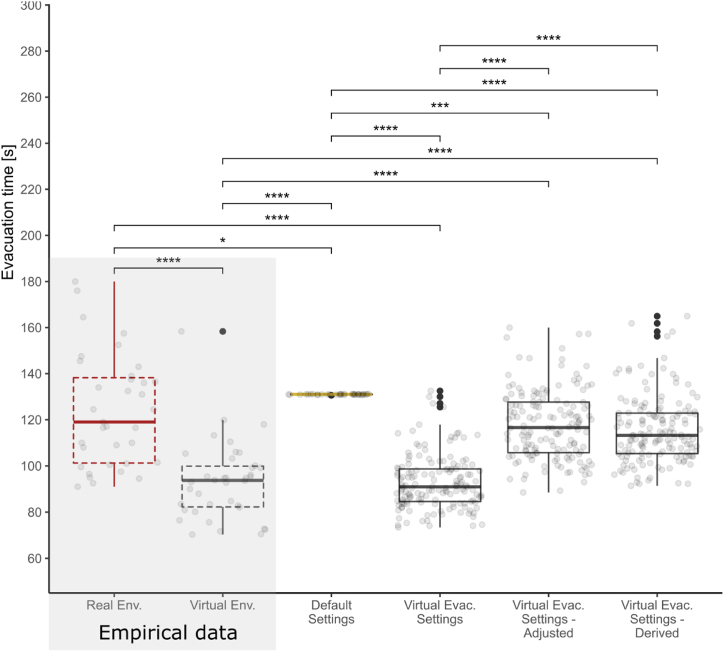
Box-plot depiction of computed evacuation times from individual evacuation models (Default Settings - DS; Virtual Evacuation Settings - VES; Virtual Evacuation Settings Adjusted - VESA; Virtual Evacuation Settings Derived - VESD). Graph is complemented with the empirical data about evacuation times from the real (RE) and virtual (VE) environments. Data were compared using Kruskal-Wallis test and Dunn’s post hoc tests, p-value adjustments (q-values) were calculated and reported due to multiple testing problem (ns: non-significant, ****: p-value <0.0001, ***: p-value = [0.0001, 0.001], **: p-value = (0.001, 0.01], *: p-value = (0.01, 0.05], outliers are defined as values > 1.5 times and <3 times the interquartile range beyond either end of the box).

The real (RE) and virtual (VE) empirical observations representing the behavior (i.e., evacuation times) of real participants [51] are depicted in the graph (Fig. 6) next to computed models involving agents, - i.e., the DS model, the VES model, the VESA model, and the VESD model. The Kruskal-Wallis test indicated significant differences in evacuation times between observations with large effect size (χ2 = 272.73, df = 5, p-value <0.001, η^2^ = 0.485). The specific differences were calculated between individual conditions and are depicted in Fig. 6, with the p-values, q-values (calculated due to multiple testing problem) and estimates shown in Table 2. The main trends are specified in more detail below.

**Table 2:** Detailed results of the mutual comparison of the resulting evacuation times between the empirical data and the models with various input settings. Evacuation times between the individual pairs (Model 1 and Model 2) were compared using post hoc Dunn’s tests with Holm’s p-value, and p-value adjustments (q-values) for between model comparison were also reported due to the high number of comparisons. (RE: Real Environment, VE: Virtual Environment, DS: Default Settings, VES: Virtual Evacuation Settings, VESA: Virtual Evacuation Settings Adjusted, VESD: Virtual Evacuation Settings Derived) (ns: non-significant, ****: p-value <0.0001, ***: p-value = [0.0001, 0.001], **: p-value = [0.001, 0.01], *: p-value = [0.01, 0.05]).

Model 1	Model 2	n1	n2	Estimate (mean ranks difference)	z value	p-value	p-value adjusted	
RE	VE	35	38	−233.917	−6.193	0.000	0.000	****
RE	DS	35	35	106.100	2.753	0.006	0.030	*
RE	VES	35	150	−243.000	−8.029	0.000	0.000	****
RE	VESA	35	150	−18.836	−0.622	0.534	1.000	ns
RE	VESD	35	150	−38.250	−1.264	0.206	0.825	ns
VE	DS	38	35	340.017	9.002	0.000	0.000	****
VE	VES	38	150	−9.083	−0.310	0.756	1.000	ns
VE	VESA	38	150	215.080	7.346	0.000	0.000	****
VE	VESD	38	150	195.667	6.683	0.000	0.000	****
DS	VES	35	150	−349.100	−11.535	0.000	0.000	****
DS	VESA	35	150	−124.936	−4.128	0.000	0.000	***
DS	VESD	35	150	−144.350	−4.770	0.000	0.000	****
VES	VESA	150	150	224.163	12.041	0.000	0.000	****
VES	VESD	150	150	204.750	10.998	0.000	0.000	****
VESA	VESD	150	150	−19.413	−1.043	0.297	0.891	ns

The output evacuation times from the computed agent models were statistically compared with empirically obtained data from both VR and real experiments [51], see Fig. 6. The statistical methods used in this paper confirmed findings of Stachoň et al. [51], that participants in VR experiment evacuated significantly quicker opposed to real experiment (RE; m = 123.93 s; med = 119.00 s; sd = 24.39 s; VE; m = 93.38 s; med = 93.73 s; sd = 17.06 s). Dunn’s post hoc tests further revealed that the evacuation times in the real condition differed significantly (p < 0.01; q-value <0.05 respectively) from the DS model (m = 130.84 s; med = 130.80 s; sd = 0.14 s). In any case, evacuation times calculated by the model seem to be overestimated, and regarding variance they do not fit the real observations. Participants in RE left the building faster on average, but with a very high variance in exit times. In fact, the total evacuation time for real hazards is determined by the last evacuees. When we consider the variance in evacuation times, the observed evidence shows that the total evacuation time as defined by complete evacuation of the building was longer in reality (RE), considering the slowest evacuees. This variance, which is lacking in the DS model, makes the original ABEM informationally poorer, since it does not offer predictions about upper limit evacuation values (or specific outliers) that are represented by the slowest evacuees. Further, models computed on the basis of the given empirical data exhibited several trends. As seen in Fig. 6, the VES model (m = 92.87 s; med = 90.95 s; sd = 11.62 s), which was generated on the basis of data from the virtual condition, differs significantly (p < 0.001; q-value <0.001) from the real condition (RE; m = 123.93 s; med = 119.00 s; sd = 24.39 s). On the other hand, both generated models that were based on VR data and corrected (i.e., the VESD model and the VESA model) corresponded to the real evacuation behavior (RE), which is an encouraging finding. Post hoc tests failed to identify any significant difference between the VESD model (m = 115.62 s; med = 113.23 s; sd = 14.47 s) and the VESA model (m = 117.80 s; med = 116.6 s; sd = 15.01 s), and neither of them significantly differed from the real condition (RE; m = 123.93 s; med = 119.00 s; sd = 24.39 s). Also, regarding values ranges, data from VESD and VESA models approximated real observation (RE). This suggests that there is correspondence between the real behavior of evacuees during evacuation drills and the simulated agent behavior computed on the corrected VR-based models. Also, both on VR data-based models (VESA, VESD) differed significantly from the Default Settings model (DS to VESA: p < 0.001, q-value <0.01; DS to VESD: p < 0.0001, q-value <0.001).

## 5 Discussion & Conclusion

The analysis of the computed models highlighted several significant trends. Most importantly, data analysis identified that participants in the experiment conducted in the real building (RE) evacuated significantly faster (p < 0.01; q-value <0.05) than agents from the Pathfinder ABEM with default settings (DS model). With respect to observed evacuation times variance this suggests that there is limited correspondence between the behavior of the people in real conditions and the agent simulation and supports previous concerns about the adequacy of the artificial parameter calculations [57]. This deficiency needs to be addressed by the use of ecologically reliable data in agent modeling in order to make better predictions about human evacuation behavior in buildings. As discussed above, conducting real evacuation drills to gather the necessary data to refine evacuation models remains complicated for several reasons [36,37]. One of the main aims of this study was to assess use of accessible VR data as an input for agent modeling. The real and VR comparisons [51] demonstrated significant differences in evacuation times between participants in real and virtual evacuations, where participants in the virtual building evacuated considerably faster than those in the real building (as seen also in Fig. 6. - Real Env. and Virtual Env.). This notion speaks for the above discussed limited validity of the raw observations made in the virtual experiments. The same difference (p < 0.001) was observed in the case of the VES model when compared to the real evacuation. This difference was expected due to the movement interface metaphor used in the virtual experiment (keyboard and mouse generated movement with default walking speed), which in the report by Stachoň et al. [51] was set as constant for the VR experiment and considerably influenced the evacuation speed of participants. This rather technical limitation has already been reflected in previous research [51] and further research is necessary in this area. In this regard, however, we support previous claims that virtual experiments *per se* should not be considered a fully adequate substitute for real evacuation drills, and only limited conclusions about evacuation behavior can be drawn on the basis of raw VR observations.

However, the identified trends in the outcomes of the corrected VR-based models (the VESA model and the VESD model) clearly support the case for the computation and possible use of agent models based on VR data, but with necessary corrections. Both the VESD and the VESA models corresponded to real evacuation behavior (RE), whereby the model simulations provided similar evacuation time and movement trajectory outcomes (see Fig. 5, Fig. 6). Since the agents in both corrected VR-based models followed trajectories that were observed in reality, the models produced evacuation time variance similar to real behavior (RE), which promotes the reliability of ABEMs established on the data from VR evacuation experiments. As demonstrated above, the VR-based agent models corrected by movement speed offer a great deal of reliability, since they approximate real evacuation processes, including movement trajectories, and offer better predictions for practical use. The data analysis failed to identify any significant difference between the VESD model and the VESA model (see Fig. 6). Regarding mean evacuation times and range, both corrected models generated from VR data corresponded to the real observation of behavior to a similar extent, which offers at least two possible methods of computation for VR-based agent models. The first method is (1) correction of the VR data on the basis of the real walking speed obtained from a specific real evacuation drill in the building under evaluation, which serves as a direct input parameter for model simulation. This method is limited by the need for a physically existing building and is therefore unsuitable in cases where the safety of a building is being assessed at the design stage. The second method is (2) post-processing correction of the VR-based model outputs. The correction can be done by theory-based or empirically based standardized coefficients (the latter was done in the case of the VESD model). This method of correction was found to be a valid, reliable, and applicable solution for making predictions about evacuation aspects of buildings, since it corresponded to real evacuation behavior. These findings represent an exciting direction for future research, whereby VR-based experiments can be adjusted for ABEMs as a cost-effective, productive, easily available, and ecologically valid tool for making predictions about the evacuation behavior of people in various types of buildings. From this perspective, VR-based ABEMs can be seen as a significant future trend, which is worthy of more detailed research. The key future direction of research is the use of interface metaphor for movement, which in VR environments allows for movement that is completely equivalent to reality. However, this will involve advanced haptic interfaces and will require considerable technological and monetary resources.

Further, VR-based models can be seen as reliable tools since they incorporate real behavioral patterns. We observed a similarity between real and agent movement insofar as the movement of agents in the simulations followed real trajectories (see Fig. 5). This provides persuasive argumentation for the equivalence of these models to reality, but for future predictions it also allows us to consider minority phenomena such as detours or getting lost during the evacuation, which demonstrably happens in reality, but is not usually incorporated into default models. These (usually minority) phenomena should be further explored with regard to critical evacuation contexts such as fractional effective dose (FED) or the slowest evacuees (which will be discussed below). Nevertheless, they greatly surpass the default models, because the original default ABEMs (DS model) did not provide any variance of the predicted evacuation times or trajectories, and therefore, in the context of this study, they were perceived to be inferior to the models based on VR empirical data. The default ABEMs lack functionality, as they do not provide, for example, the upper quantiles (e.g., preset at 95%), since they only generate measures of central tendency. Computed corrected VR-based models (the VES model and the VESA model) provided information involving the distribution of evacuation times including potential outliers (i.e., extremely slow people), whose safety it is also necessary to secure. Statistically speaking, evacuees present in the third quartile of evacuation times (the longest) represent the most vulnerable population, since they are last to exit the building, so evacuation procedures should be particularly focused on this group. In order to do this, empirical observations of evacuation drills are necessary to capture potential anomalies and outliers, and in this respect, VR can significantly promote their availability. Moreover, a significant benefit of VR experiments for practical applications is the controllable, safe, and dynamic modulation of the environment for empirical testing. The use of dynamic models to study, for example, fires [40] or the spread of toxins can help to identify deviations in the behavior patterns of individuals that differ from ideal or assumed movement trajectories (i.e., passage route), and constitute an area for future research. These movement deviations may cause delays and outlying values in decision-making times specifically in relation to critical points in the area of the researchers’ interest (e.g., blocked passage etc.). Such deviations in the evacuation process may have a significant influence on exposure to toxic substances or temperature and thus on the final fractional effective dose (FED).

VR-based models preserve the above-discussed advantages of VR evacuation experiments, such as cost-effectiveness, speed, dynamic modulation, accessibility, and data logging options [22]. More importantly, they can also be applied during the planning phase of buildings [37]. Regardless of the above mentioned, VR technologies still possess several limitations [40,52,53], which should be kept in mind when designing, conducting or interpreting VR-based research. As for the primary limitations of VR applications in this field, they remain the commonly reported issues such as cybersickness, i.e., the feeling of nausea when using virtual displays such as HMDs, especially when moving, which causes considerable experiment mortality and therefore necessitates larger sample sizes. Moreover, the model data optimization is determined by the specific nature of the research sample, and it is necessary to consider the fact that such ABEM predictions apply primarily for the studied populations, and they reflect the specific density and structure of the studied population. With regard to the above, in order to follow principles of external validity, any further empirical measurements made for the purpose of potential application in real practice should include an adequate representation of the target population and should consider its specific size. Regardless of the above-mentioned limitations, we conclude that the results of this study support the viability of reliable and ecologically valid empirical inputs from virtual experiments for agent-based evacuation modeling.

A promising area of follow-up research is the exploration of multi-agent or crowd behavior [44]. In the case of this study, the evacuation behavior of individuals was captured and used for the follow-up single agent simulations. However, virtual experiments, by their very nature, offer great possibilities for concurrent and massive-scale data collection from large numbers of participants even in distant places all around the world at a specific time. The specific approach of this study can potentially be adopted and implemented for ABEMS in a similar way for the groups of evacuees. However, in this regard, specific research on assessing the possible differences in crowd behavior under real and virtual conditions certainly needs to be further explored in order to provide clear arguments for VR use in crowd agent-based evacuation modeling. In any case, the application of multi-agent modeling optimized by the parameters from empirical experiments remains an exciting direction of future research.

## Author contribution statement

Vojtěch Juřík; Dajana Snopková: Conceived and designed the experiments; Analyzed and interpreted the data; Wrote the paper.

Ondřej Uhlík: Conceived and designed the experiments; Performed the experiments; Analyzed and interpreted the data; Wrote the paper.

Ondřej Kvarda; Tomáš Apeltauer: Contributed reagents, materials, analysis tools or data; Wrote the paper.

Jiří Apeltauer: Conceived and designed the experiments; Contributed reagents, materials, analysis tools or data; Wrote the paper.

## Funding statement

This work was supported by 10.13039/501100002969Technologická Agentura České Republiky [TL02000103].

## Data availability statement

Data will be made available on request.

## Declaration of interest’s statement

The authors declare no competing interests.

## Additional information

Supplementary content related to this article has been published online at [URL].

## Appendix A Supplementary data

Multimedia component 1 ABEM with default settings – First person view of evacuation using the official marked exit

Multimedia component 2 ABEM with default settings – Perspective view of evacuation using the official marked exit

Multimedia component 3 ABEM with Real Evacuation settings – First person view of evacuation using an alternative exit

Multimedia component 4 ABEM with Real Evacuation settings – Perspective view of evacuation using an alternative exit

Multimedia component 5 ABEM with Virtual Environment Evacuation settings – First person view of evacuation using an alternative exit

Multimedia component 6 ABEM with Virtual Environment Evacuation settings – Perspective view of evacuation using an alternative exit

Multimedia component 7 ABEM with Virtual Environment Evacuation settings and walking speeds adopted from Real Evacuation – First person view of evacuation using an alternative exit including a detour to the elevator

Multimedia component 8 ABEM with Virtual Environment Evacuation settings and walking speeds adopted from Real Evacuation – Perspective view of evacuation using an alternative exit including a detour to the elevator

## Footnotes

1 In this text, we use the term “VR experiments” instead of “VR simulations” to avoid potential confusion with “agent simulations”.
